# False Positive Transit Time Flowmetry Graft Failure in Multivessel Coronary Spasm following Off-Pump Coronary Artery Bypass Grafting

**DOI:** 10.1155/2017/3186047

**Published:** 2017-05-28

**Authors:** George Kassimis, George Krasopoulos

**Affiliations:** ^1^Cardiology Department, Gloucestershire Hospitals NHS Foundation Trust, Cheltenham, UK; ^2^Oxford Heart Centre, Oxford University Hospital, Oxford, UK

## Abstract

Intraoperative Transit Time Flowmetry is currently recommended to assess graft patency during coronary artery bypass grafting (CABG), especially in presence of haemodynamic instability or inability to wean the patient from cardiopulmonary bypass, new regional wall motion abnormalities, or significant ventricular arrhythmias. The VeriQ™ system is one of the currently available systems, which detects imperfections that may be corrected by graft revision. In this case report, multivessel coronary spasm (CS) post-CABG interferes with these intraoperative parameters misleading initially into false results. Cardiac surgeons should bear in mind the limit of VeriQ in distinguishing between graft failure and CS. Angiography may be considered in patients with decreased graft flow despite revision of anastomosis and vasodilatory treatment for the definitive diagnosis.

## 1. Introduction

Intraoperative Transit Time Flowmetry (TTFM) is currently recommended to assess graft patency during coronary artery bypass grafting (CABG), especially in presence of haemodynamic instability or inability to wean the patient from cardiopulmonary bypass, new regional wall motion abnormalities, or significant ventricular arrhythmias. TTFM is effective in detecting imperfections that may be corrected by graft revision. This may reduce the incidence of graft occlusion and may reduce perioperative morbidity and mortality [1].

The VeriQ system (MediStim ASA) is one of the currently available systems, which uses specially designed probes, which deliver a bidirectional ultrasound beam across a target vessel, and the system analyses the returning signal to calculate the blood flow through the vessel at a default filter setting of 20 Hz. A real-time flow waveform is displayed, together with the mean graft flow (MGF) in ml/min and derived values such as pulsatility index (PI). This information can be used to determine whether flow through the graft and its anastomoses is acceptable or not. MGF ≤ 20 ml/minute and/or a pulsatility index ≥ 5 predict technically inadequate grafts that need immediate revision before leaving the operating theatre. Coronary spasm post-CABG can potentially interfere with these intraoperative parameters misleading into false results [2].

## 2. Case Report

**Figure 1:**
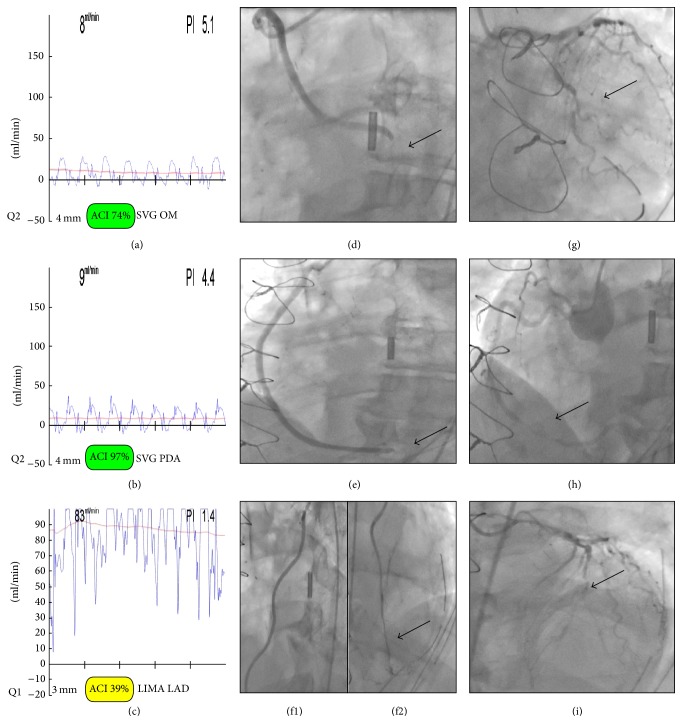
The VeriQ will display the Doppler spectrum at the default 5 seconds sweep rate as soon as a probe is connected, as shown in the figure. In Figures 1(a)–1(c) there is a flow-time curve of SVG to OM, SVG to PDA, and LIMA to LAD, respectively. After completion of all three grafts, there was good MGF in the arterial but not in the vein grafts. TTFM measurements with a mean arterial pressure of 80 mmHg were as follows: LIMA to LAD: MGF 83 ml/min and PI 1.4; LSV to OM: MGF 8 ml/min and PI 5.1; LSV to PDA: MGF 9 ml/min and PI 4.4 (Figures 1(a)–1(c)). An urgent angiography confirmed the patency of all three grafts with wide spread evidence of spasm to native coronary arteries (Figures 1(d)–1(i)). The arrows refer to spasm.

A 71-year-old diabetic man was admitted to the Oxford Heart Centre within 48 hours from an episode of angina at rest. Coronary angiogram demonstrated severe left main stem stenosis and three vessels coronary artery disease, with a preserved left ventricular systolic function. The patient underwent off-pump CABG using skeletonized left internal mammary artery (LIMA) to the LAD and long saphenous vein (LSV) grafts to the OM and PDA arteries. After completion of the all three grafts, there was excellent flow in the arterial but not in the vein grafts. TTFM measurements with a mean arterial pressure of 80 mmHg were as follows: LIMA to LAD: MGF 83 ml/min and PI 1.4; LSV to OM: MGF 8 ml/min and PI 5.1; LSV to PDA: MGF 9 ml/min and PI 4.4 (Figures 1(a)–1(c)).

At the end of the procedure the patient developed significant ECG ST depression. The sternotomy was reopened and all grafts were revised. LIMA to LAD was found to be widely patent but both vein grafts had minimal flow with no distal run-off. The patient's conditions were stabilised with the use of inotropes and intra-arterial balloon pump (IABP). An urgent angiography confirmed the patency of all three grafts with wide spread evidence of spasm to native coronary arteries (Figures 1(d)–1(i)).

The patient was started on glycerine trinitrate infusion; the adrenaline and noradrenaline were weaned successfully with complete ST resolution and he was discharged home on the 8th postoperative day with fully controlled diabetes and on 10 mg of isosorbide mononitrate, once daily, as a secondary prevention of coronary spasm for 3 months.

## 3. Discussion

The intraoperative assessment of graft flow with TTFM and Doppler techniques has been largely accepted and is recognized by 2014 ESC-EACTS guidelines on myocardial revascularization. TTFM is a useful tool towards the right assessment of graft patency and flow but in rare occasions can be misleading and it may fail to detect the real cause of poor flow through the grafts. Furthermore, interpretation can be challenging in sequential grafts and T-grafts [3–7].

The assessment of the graft patency using VeriQ during coronary spasm can be misleading and can provide, as in our case, false positive results, which if not promptly identified can be the cause of increased mortality and morbidity.

Perioperative CS is a potentially, life-threatening condition, which if not early detected and successfully treated can be catastrophic. Its prevalence during CABG is reported to range from 0.8 to 1.3% and has been documented in patients undergoing on- and off-pump CABG. Main risk factors for CS are smoking, hyperlipidaemia, advanced age, diabetes, chronic renal impairment and factors related to anaesthesia such as insufficient general anaesthesia, stimulation of the vagal nerve, hyperventilation, hypoxia, and use of large doses of catecholamines [8].

The case described here represents also an extraordinary example of how, despite a generalized CS, the LIMA graft was somehow protected. It is well known how the particular structure of LIMA wall makes it less prone to spasm, even though not immune, compared to other arterial grafts. Endothelial integrity and its pronounced capacity of releasing nitric oxide may also play a significant role in preventing LIMA spasm. In this particular case, the LIMA was harvested in skeletonized fashion, questioning whether minimal thermic injury could effectively contribute to protecting the arterial graft against vascular spasm [9, 10].

Pharmacological treatment of CS includes the administration of nitrates and calcium antagonists. Cardiovascular support devices such as IABP can also be very helpful in managing the acute phase of CS. If medical therapy and IABP are not proven to be sufficient, extracorporeal membrane oxygenation may also become necessary to salvage the patient [9, 11].

## 4. Conclusion

This case describes the occurrence of CS following off-pump CABG in a male patient with a poor controlled diabetes. TTFM measurements suggested impaired vein graft glow, but subsequent coronary angiography confirmed graft patency with significant CS.

Cardiac surgeons should bear in mind the limit of VeriQ in distinguishing between graft failure and CS. Angiography may be considered in patients with decreased graft flow despite revision of anastomosis and vasodilatory treatment for the definitive diagnosis. The treatment for CS during CABG remains debated; time and vasodilators seem to be the most appropriate way of addressing this rare but dangerous clinical condition.
